# How to strengthen primary health care? An exploratory study on the policy of vertical integration of high-quality medical resources based on symbiosis theory

**DOI:** 10.3389/fpubh.2025.1578712

**Published:** 2025-05-14

**Authors:** Linyan Zhao, Jie Du, Wenhao Liu, Qun Xu, Yuhui Zhang

**Affiliations:** ^1^School of Public Health, Shandong Second Medical University, Weifang, China; ^2^Second People’s Hospital of Weifang, Weifang, China; ^3^Binzhou Medical University, Yantai, China; ^4^Hainan Provincial Health Commission, Haikou, China

**Keywords:** primary health care, vertical integration, medical resource, symbiosis theory, health policy

## Abstract

**Background:**

The Vertical Integration of High-Quality Medical Resources (VI-HQMR) is a strategy of medical resource reallocation. It is the key to strengthen primary health care (PHC) and build an integrated delivery system (IDS). It contributes to the Sustainable Development Goals (SDGs) of universal health coverage (UHC) set out by the World Health Organization (WHO). In order to VI-HQMR, countries around the world have carried out many beneficial explorations. However, our understanding of the importance of clarifying the internal logical from policy perspective in the VI-HQMR is limited. This study aims to develop a theoretical model from the symbiotic perspective to improve the strategy of VI-HQMR.

**Methods:**

Policies related to the VI-HQMR were retrieved for exploratory research. The texts and entries were coded according to the four elements of symbiosis theory, the first-level categories and their variables were mined, and the occurrence frequency was used as the main indicator for thematic clustering.

**Results:**

A total of 609 policies were retrieved, among which 1,072 entries mentioned VI-HQMR. Results showed that the VI-HQMR included 482 symbiotic units, 549 symbiotic models, 383 symbiotic environments and 96 symbiotic interfaces. Secondary and above public hospitals and PHC institutions are the most important symbiotic units. Medical alliances are the most important symbiotic model. The symbiotic environment includes policy, technology and economics. The vertical integration of human resources is the main symbiotic interface.

**Conclusion:**

The VI-HQMR is still in the initial exploration stage. The symbiotic model is changing from parasitism to the commensalism. To achieve the optimal mutualism model, we need to work hard from the symbiotic environment. Health administrative department should coordinate with other relevant departments to introduce special policies to support the VI-HQMR. Through opening the way for promotion, financial incentive, and informationization assistance, improve the enthusiasm of urban hospitals.

## 1 Introduction

Universal health coverage (UHC) is one of the health-related Sustainable Development Goals (SDGs) established by the World Health Organization (WHO). Primary health care (PHC) is the cornerstone of UHC and plays a crucial role in promoting health equity. To strengthen PHC worldwide, WHO has identified three strategic areas of work, one is promoting PHC renewal through policy leadership, advocacy and strategic partnerships with governments, non-governmental organizations, civil society organizations, development partners, UN sister agencies, donors, and other stakeholders at global, regional and country levels. In the past decade, recent progress in increasing coverage has slowed, especially the COVID-19 pandemic has impacted the original order. In order to better rebuild, the prominent problem of unbalanced allocation of medical resources must be solved. Medical resources are fundamental to achieving high-quality and efficient PHC. Both developed and developing countries face challenges related to the imbalanced allocation of medical resources (1–5). High-quality medical resources are often concentrated in urban areas or a few medical institutions, while medical resources are relatively scarce in rural and remote areas. Especially significant disparities between regions hinder the quality and efficiency of the PHC, this is detrimental to achieve UHC, promote health equity.

In China, the issue of unequal distribution of medical resources between urban and rural areas is particularly acute, resulting in poor access to medical resources leads to PHC remaining the weakest link in the health system. As China’s medical and health system reform progresses, the overall availability of PHC resources has increased, and regional disparities in allocation have gradually diminished. However, as equity in PHC improves, efficiency has emerged as a new concern. The Chinese government has begun to advocate for the development of a high-quality, efficient, and vertically integrated delivery system (IDS). This shift necessitates an enhancement of PHC, which entails a greater need for funding, skilled professionals, and advanced technologies. Additionally, the uneven distribution of medical resources within regions (4, 6) remains a significant barrier to improving PHC capacity.

To address this issue, China has initiated the promotion of Vertical Integration of High-Quality Medical Resources (VI-HQMR). This approach aims to leverage the high-quality medical resources of advanced medical institutions and regions to enhance the capacity of PHC capacity. VI-HQMR involves redistributing high-quality medical resources from urban areas to rural regions or from large medical institutions to PHC facilities (7). China has introduced several policies to support VI-HQMR, including the establishment of medical alliances, the implementation of a hierarchical diagnosis and treatment system (8), and the provision of telemedicine services (9). Additionally, various countries worldwide have adopted measures to promote VI-HQMR, such as the Kaiser Permanente model in the United States (10), the Trust model in the United Kingdom (11), the Disease Management Project in Germany (12), and the two-group model in Singapore (13).

Currently, research on the VI-HQMR mainly includes primarily examines the impact of vertical integration on various operational indicators of medical institutions. These indicators include readmission rates (14), patient satisfaction (15, 16), access to surgical nursing (17), the volume of inpatient and outpatient services (18), and the quality of care for chronic diseases such as hypertension and diabetes (19). Additionally, the quality of healthcare services (20) and the evaluation of medical personnel within medical alliances (21) are also considered. In terms of vertical integration of medical resources, the focus is on high-quality medical talent, services (22), and technological resources (23) that facilitate this integration. The specialties involved in this research include emergency (24), nursing (25), oncology (26), pediatrics (27), infectious diseases (28), and rehabilitation medicine (29). Overall, the research on VI-HQMR primarily concentrates on evaluating the effectiveness of implementation among participants and has yet to establish a unified, scientific, and universally applicable evaluation index system.

The current research focuses on improving the operational efficiency of PHC institutions through the VI-HQMR framework; however, several issues remain to be addressed. First, analyzing the logic of policy implementation is essential to ensure that policies are executed effectively and that desired objectives are met. Currently, there is a lack of research on the internal logic of VI-HQMR, and the results of effect evaluations cannot be translated into practical guidance for policy optimization. Second, there is no comprehensive summary of the participating institutions, resource disciplines, and specific forms of VI-HQMR, indicating a need for thorough research on this initiative. Finally, there is a gap in research regarding the vertical integration mechanism of high-quality medical resources from a systematic perspective.

The core of the VI-HQMR is to foster effective collaboration among medical and health institutions at all levels, with the ultimate goal of achieving mutual development for all parties involved. Consequently, we adopt the theory of symbiosis as the foundational framework for this study. The term “symbiosis” originates from the field of biology and is utilized to explore the material connections between different organisms. It encompasses three key elements: the symbiotic unit, the symbiotic model, and the symbiotic environment. These elements collectively form a symbiotic interface. The symbiotic unit serves as the fundamental building block of symbiosis, describing the compatibility of the internal properties of the symbiotic unit and its changing qualitative parameters, which are essential for determining the existence of a symbiotic relationship. The process of symbiosis among these units involves a consistent alignment of mass parameters. The symbiotic model comprises two key components: symbiotic behavior and the degree of symbiotic organization. Symbiotic behavior encompasses the interactions among symbiotic units, which include parasitism, commensalism, and mutualism. The degree of symbiotic organization is categorized into four types: point symbiosis, intermittent symbiosis, continuous symbiosis, and integrated symbiosis. The primary distinction among these types lies in the nature of cooperation between symbiotic unit is brief at a specific moment, intermittent, or continuous over a defined period. Integrated symbiosis represents the highest level of cooperation. The symbiotic environment refers to the external conditions that facilitate the existence and development of the symbiotic model, with all factors outside the symbiotic unit contributing to this environment (30, 31). Symbiosis theory can be employed to analyze the interactions between different agents within a system, as well as the dynamic relationships between these agents and their environment (32).

Symbiosis theory was initially developed to address questions related to biological fields, such as the origin of cells (33, 34). Currently, this theory has been applied to research on resource synergy, including interactions between urban and rural areas (35), as well as the interconnections among water, energy, and food (36, 37). Furthermore, several studies have explored the application of symbiosis theory in the analysis of industrial policy (38).

The research field of symbiosis theory continues to expand, encompassing not only biology but also botany, genetics, agriculture, management, and other disciplines. Effective health policy typically necessitates coordination and collaboration. This study applies the symbiosis theory to study the policies related to the VI-HQMR, which is an innovative application in the field of health service management. By developing a theoretical model of the VI-HQMR, this research aims to offer insights for related studies in other countries.

This study innovatively applies the logical framework of symbiosis theory to analyze national and provincial policy texts related to the VI-HQMR. On one hand, it describes the overall landscape of these policy texts in China, including the number of releases, historical evolution, and temporal distribution. On the other hand, we analyze the internal logic of the VI-HQMR, which involves clarifying the participants, summarizing the cooperation model, identifying the symbiotic environment necessary for this work, outlining the symbiotic interface, and constructing a theoretical model of the VI-HQMR. Our aim is to provide a theoretical reference for countries worldwide to strengthen PHC, promote health equity, and achieve UHC.

## 2 Materials and methods

### 2.1 Data collection

Firstly, in order to ensure the authority and representativeness of the policy documents, this study took the official websites of the National People’s Government and the National Health Commission as the initial search entry points, and conducted searches using keywords such as “resource sinking” “vertical” “mobile” “medical resources” “support” “help” and “alliance” to initially obtain the policies related to the VI-HQMR at the national level, and to interpret the core content of the VI-HQMR. Secondly, further complement “regional medical center” “specialist league” “medical alliance” “medical group” as keywords. Search the relevant policies at the provincial level from the official websites of 31 provinces (autonomous regions and municipalities directly under the Central Government) except Hong Kong, Macao and Taiwan. Finally, in order to ensure the comprehensiveness of the retrieval, two personnel familiar with VI-HQMR and policy research conducted a comprehensive retrieval, cleaning and processing of the policy documents released on the national and provincial (autonomous region or municipality directly under the Central Government) official websites, respectively. The research team established four selection criteria: (1) policies issued by the People’s Government and the Health Commission; (2) policies containing relevant contents on the VI-HQMR; (3) documents that included regulations, notices, plans, measures and opinions, but excluded policy interpretation documents; (4) removed the forwarded documents and invalid documents. After the above steps, the policy documents included in this study consist of 46 national policy documents and 563 provincial policy documents, totaling 609 policy documents. Among the 609 policy documents, the earliest policy document was introduced on June 18, 2009, and the latest policy document was introduced on October 31, 2024. The time span of the policy documents included in this study is nearly 15 years.

### 2.2 Research methods

#### 2.2.1 Exploratory research

Exploratory research is a fundamental method in social science research. At the initial stage of the research, when the researcher is not clear about the nature, scope, and related factors of the research problem, this method can be employed to gain further direction. At present, there have been many researches using exploratory research methods, which are mainly used to explore the views of a certain group on an event (39–43) and the influencing factors of an event (44–46). In policy analysis, this method is used to explore policy formulation and evaluation (47, 48), policy evolution trends (49, 50), influencing factors of policy implementation (51, 52), and policy implementation obstacles (53, 54), which can lay a good foundation for policy optimization. Therefore, this study conducted an exploratory analysis of the VI-HQMR policies introduced at both national and provincial levels, coding the policy documents and entries with the aim of developing a theoretical model of the VI-HQMR to provide certain references for the subsequent policy optimization.

#### 2.2.2 Content analysis

Content analysis is a research method to objectively, systematically and quantitatively describe the content of communication (55), which is often employed to analyze various forms of information, including text, images, audio, and video. By classifying and coding these contents and describing their characteristics quantitatively, content analysis can reveal underlying patterns, trends, and themes. It is widely applied in the analysis of policy texts (56–61). This study applied content analysis to examine the coded policy entries, extracting information on symbiotic units, symbiotic models, symbiotic environments, and symbiotic interfaces of VI-HQMR, while also counting their frequency. Subsequently, the themes were clustered, and a cooperation network among the symbiotic units was established to accurately reflect the interconnections among each unit and elucidate the internal logic of VI-HQMR in China.

### 2.3 Research design

This study consists of two main steps: coding and classification, followed by theoretical model construction. The specific steps are outlined below.

#### 2.3.1 Coding and classification

In this section, policy documents and entries related to VI-HQMR are coded. Additionally, the analysis unit and construction category are established, and the relevant content is extracted from the policy entries based on the construction category, followed by encoding the content.

#### 2.3.2 Policy text coding

A total of 1,072 entries were extracted from 609 policy documents, which included 62 entries at the national level and 1,010 entries at the provincial level (encompassing autonomous regions and municipalities directly under the central government). Excel 2016 was utilized for data cleaning and coding of the retrieved policy documents. The high-quality vertical integration entries of medical resources extracted from these documents were coded using the format “hierarchy code - file code - entry code.” For example, the code of the country is “00,” the code of the *Notice of The General Office of the State Council on the issuance of the “14th Five-Year Plan” national Health Plan* is “29”, and the code of “Promoting the sinking of pharmaceutical care in medical joint bodies, clinical pharmacists guiding primary medical and health institutions to improve the level of rational drug use, focusing on providing drug guidance for chronic disease patients who have contracted services” is “6-2.” Therefore, the code for this entry is “00-29-6-2”.

#### 2.3.3 Entry content encoding

The first step is to identify the analysis unit and establish categories. Each policy entry is considered as an analysis unit, while the elements of symbiosis theory are classified as first-level categories. This study includes 1,072 policy entries from 609 policy documents. The symbiosis theory encompasses the symbiotic unit, symbiotic model, and symbiotic environment. Consequently, this study comprises a total of 1,072 analysis units and has preliminarily identified three first-level categories. Next, 62 policy entries from 46 national-level policy documents were included in the test database. The corresponding content of the first-level categories in these 62 policy entries was refined to determine the second-level categories. Following the test, the complementary symbiotic interface was classified as a first-level category, resulting in the identification of four first-level categories. We gained an initial understanding of the second-level categories corresponding to these four first-level categories in preparation for formal content coding.

The next step is to encode the content. Content coding is performed by two trained coders to ensure the quality of the coding process. The DiVoMiner online text data mining and analysis platform was utilized to code 1,072 policy entries, with the answer type for the first-level category being text. In the initial round, both coders assigned second-level categories corresponding to the four first-level categories. Based on quality monitoring results, the coding outcomes for similar categories were standardized; for instance, “medical staff “human resources and “experts” were all normalized to After the second round of coding, the quality monitoring procedures were repeated until the third round was completed, resulting in consistent coding outcomes from both coders. According to the logical framework of symbiotic theory, 697 entries contained symbiotic units, 746 entries contained symbiotic modes, 442 entries contained symbiotic environments, and 905 entries contained symbiotic interfaces, all selected from the 1,072 policy entries. Based on the results of the first-level category construction and content coding, the options under each first-level category were removed and counted to derive the second-level categories. The symbiotic unit comprises 482 s-level categories, while the symbiotic model includes 549 s-level categories. The symbiotic environment offers 383 options derived from 4 s-level categories, and the symbiotic interface consists of 96 s-level categories.

The research questions of this study can be addressed by analyzing the coding results: (i) What are the symbiotic units of VI-HQMR? (ii) How are the symbiotic units related to one another? (iii) What is the symbiotic environment that supports the symbiotic model? (iv) What are the symbiotic interfaces of the VI-HQMR?

#### 2.3.4 Construction of theoretical model

The primary objective of this study is to develop a theoretical model of VI-HQMR grounded in symbiosis theory. Based on the coding results, we will create a custom dictionary and eliminate stop words. The theoretical model of VI-HQMR is constructed through topic clustering. During the construction phase of the theoretical model, the processes and analyses of each first-level category are conducted independently. Regarding symbiotic units, we establish a cooperation network that highlights the main symbiotic units and the flow direction of high-quality medical resources. In terms of symbiosis models, we employ thematic analysis to cluster the policy, technology, and economic environment, extracting the corresponding core elements. At the symbiotic interface, we identify the interface with the highest frequency based on word frequency analysis, which provides a more intuitive representation of the elements of VI-HQMR. Following an independent analysis of each component of symbiosis theory, we utilized mapping software to integrate these elements and construct a theoretical model of VI-HQMR based on policy.

## 3 Results

### 3.1 Overview of vertical integration of high – quality medical resource policies

#### 3.1.1 Historical evolution of policies at the national level

Policies at the national level serve as essential guidance and compliance frameworks for all regions to implement their work effectively. Analyzing the historical evolution of these national policies provides a comprehensive understanding of the advancement process of the VI-HQMR. The VI-HQMR is a significant initiative aimed at deepening the reform of China’s medical and health system. It has been 15 years since China initiated a new round of medical and health system reform in 2009, during which a total of 46 policy documents related to the VI-HQMR have been introduced at the national level, resulting in the extraction of 62 policy entries.

**Figure 1:**
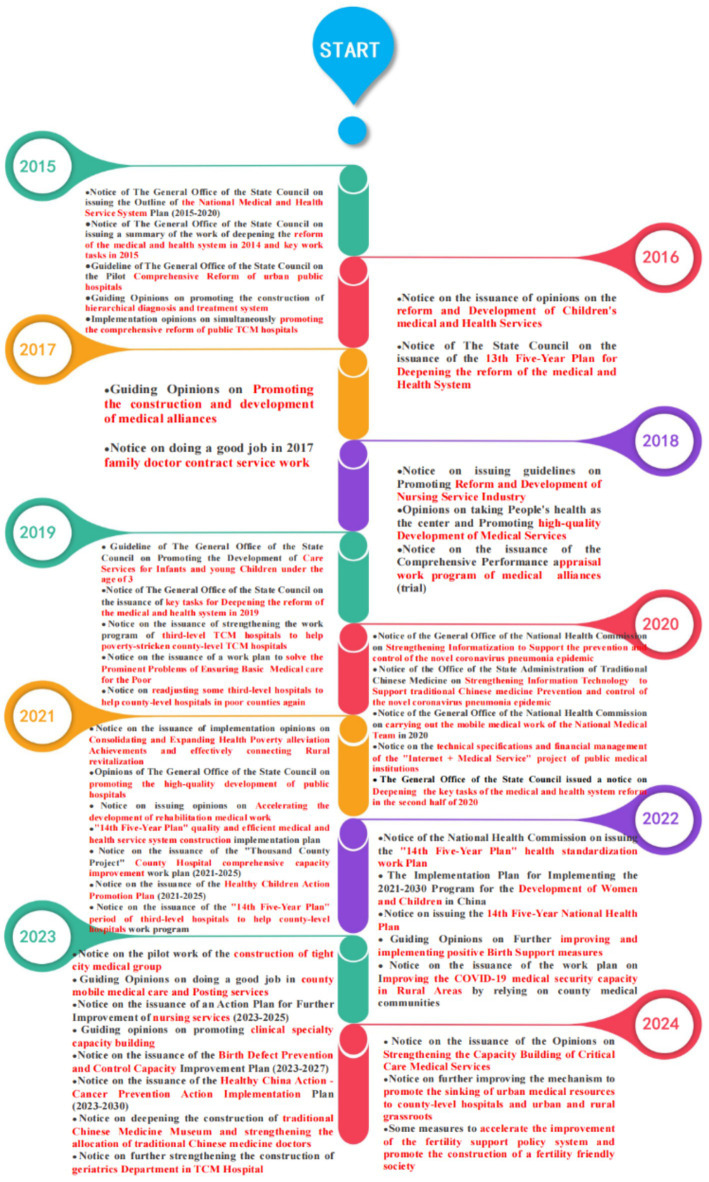
The historical evolution of vertical integration policies of high-quality medical resources at the national level.

In 2015, the General Office of the State Council introduced *the Notice of The General Office of the State Council on Printing and Distributing the Outline of the National Medical and Health Service System Planning (2015–2020)*, which for the first time proposed the concept of the VI-HQMR. This initiative required public hospitals to assist and guide primary medical and health institutions with which they have a division of labor and cooperation. Beginning in 2015, national policies were introduced to promote the VI-HQMR. From 2015 to 2024, a total of 5, 2, 2, 4, 5, 5, 7, 5, 8, and 3 policy documents were introduced, respectively. The distribution and main themes of these policy documents are illustrated in Figure 1. In 2023, the number of policy documents introduced at the national level reached an all-time high, addressing various themes such as the establishment of integrated urban medical groups, itinerant county healthcare services, nursing services, the development of clinical specialty capacities, strategies for the prevention and treatment of birth defects, implementation plans for cancer prevention and treatment, the establishment of traditional Chinese medicine stations, and the development of geriatrics departments within traditional Chinese medicine hospitals. By 2024, China introduced a *Notice aimed at further enhancing the mechanism for promoting the allocation of urban medical resources to county-level hospitals and grassroots urban and rural healthcare facilities*. This document marks the first dedicated policy on the VI-HQMR. It outlines the direction for VI-HQMR through five key aspects: (1) Urban hospitals supporting county-level hospitals. (2) Urban hospitals assisting community healthcare centers. (3) Hospitals above the county level providing support to township health centers and village clinics. (4) The implementation of rural itinerant medical services. (5) Utilizing information technology to connect medical institutions across all levels. The overarching goal is to gradually achieve the VI-HQMR, facilitating the flow of resources from urban centers to county-level hospitals.

#### 3.1.2 Quantitative distribution of policies at the provincial level

**Figure 2:**
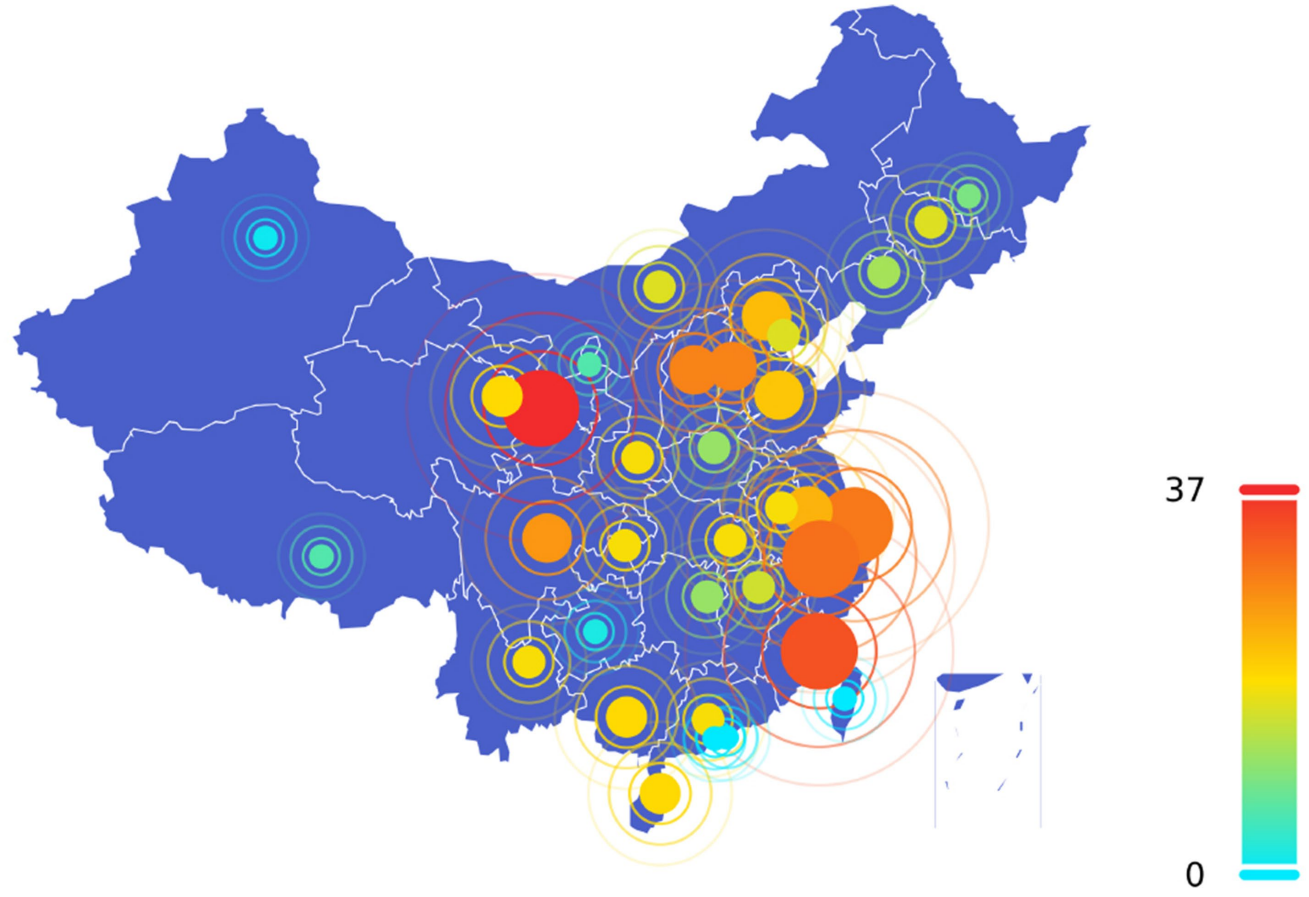
Quantity distribution of vertical integration policies of high-quality medical resources at provincial level. The dot size corresponds to the number of policies issued per province, with larger dots indicating higher policy counts. The color of each dot also indicates the number of policies. As shown in the legend on the right side of the figure, from blue to yellow, green and red represent a gradual increase in the number of policies.

At the provincial level, the first policy related to the VI-HQMR was introduced by Shandong Province in 2009. This policy focused on promoting the construction of a basic medical security system for urban areas, requiring the exploration of methods for pooling general outpatient expenses under basic medical insurance. It emphasized the reliance on community health services to facilitate access to designated medical institutions. From 2009 to 2024, the number and distribution of policies related to the VI-HQMR introduced by the 31 provinces (autonomous regions and municipalities directly under the Central Government) in China are illustrated in Figure 2. China’s policy of VI-HQMR mainly comes from the eastern regions. Ten provinces (autonomous regions and municipalities directly under the central government) in eastern China have issued 252 policies for VI-HQMR, accounting for 41.4 percent of the total. Among them, 22 were in Beijing, 16 in Tianjin, 41 in Hebei, 21 in Shandong, 23 in Jiangsu, 29 in Shanghai, 30 in Zhejiang, 33 in Fujian, 18 in Guangdong, and 19 in Hainan. The top five provinces (autonomous regions and municipalities directly under the Central Government) with the largest number of policy documents are Gansu Province, Fujian Province, Zhejiang Province, Shanghai and Hebei Province. With the exception of Gansu Province, which is situated in the western part of China, the other four provinces are located in the economically developed and resource-rich eastern region. Gansu Province introduced 37 policy documents, from which 77 entries were extracted. In 2021, Gansu Province released seven related policy documents, focusing primarily on maternal and child health, tumor diagnosis and treatment, the development of traditional Chinese medicine, and the high-quality development of public hospitals. Excluding Hong Kong, Macao, and Taiwan, Xinjiang introduced the fewest policies, with only one document addressing traditional Chinese medicine in the context of maternal and child health. Guizhou Province, Ningxia Hui Autonomous Region, Tibet Autonomous Region, and Heilongjiang Province also introduced a limited number of policies.

#### 3.1.3 Time distribution of policies at the provincial level

**Figure 3:**
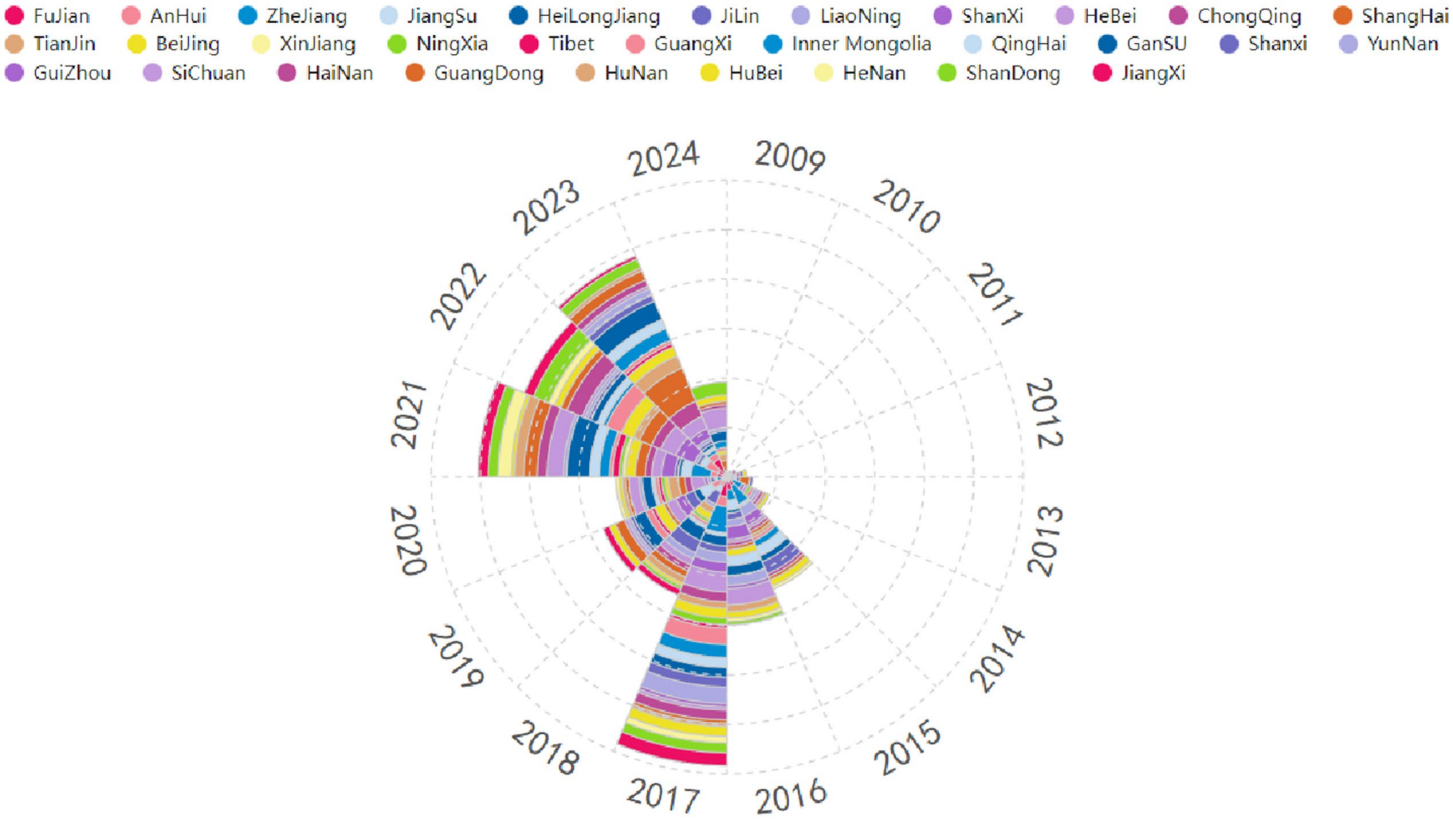
Time distribution of vertical integration of high-quality medical resource policies at the provincial level. (1) The different colors represent different provinces (autonomous regions or municipalities directly under the Central Government) of China. The provinces (autonomous regions or municipalities directly under the Central Government) represented by each color are detailed in the legend above the picture. (2) The area occupied by each color represents the number of policies for the VI-HQMR issued by the corresponding province (autonomous regions or municipalities directly under the Central Government) within a specific period of time. The larger the area occupied, the more policies were issued by this province during this period of time, the smaller the area occupied, the fewer policy documents were issued.

To visually illustrate the temporal trends of policies related to the VI-HQMR introduced at the provincial level in China from 2009 to 2024, we present Figure 3 to quickly identify the phased characteristics of the VI-HQMR. Overall, the number of policies introduced at the provincial level has shown an upward trajectory. From 2009 to 2017, the number of policies gradually increased, peaking in 2017 with a total of 88 policies introduced. However, from 2018 to 2023, the number of policies exhibited fluctuations. Notably, during the three-year period from 2021 to 2023, there was a significant increase in the introduction of policies related to the VI-HQMR at the provincial level. The temporal distribution of policies at the provincial level differs from that at the national level, with the fluctuations in the number of provincial policies being significantly more pronounced. In 2017, Zhejiang Province introduced the highest number of policies related to the VI-HQMR, totaling 8. In 2021, Gansu Province led with 7 policies. Additionally, Shanxi Province and Shanghai recorded the highest numbers of policy introductions in 2022 and 2023, respectively, with Shanxi Province introducing 3 policies in 2022 and Shanghai introducing 11 policies in 2023.

### 3.2 Internal logic analysis of vertical integration of high-quality medical resources

#### 3.2.1 Symbiotic unit

**Figure 4:**
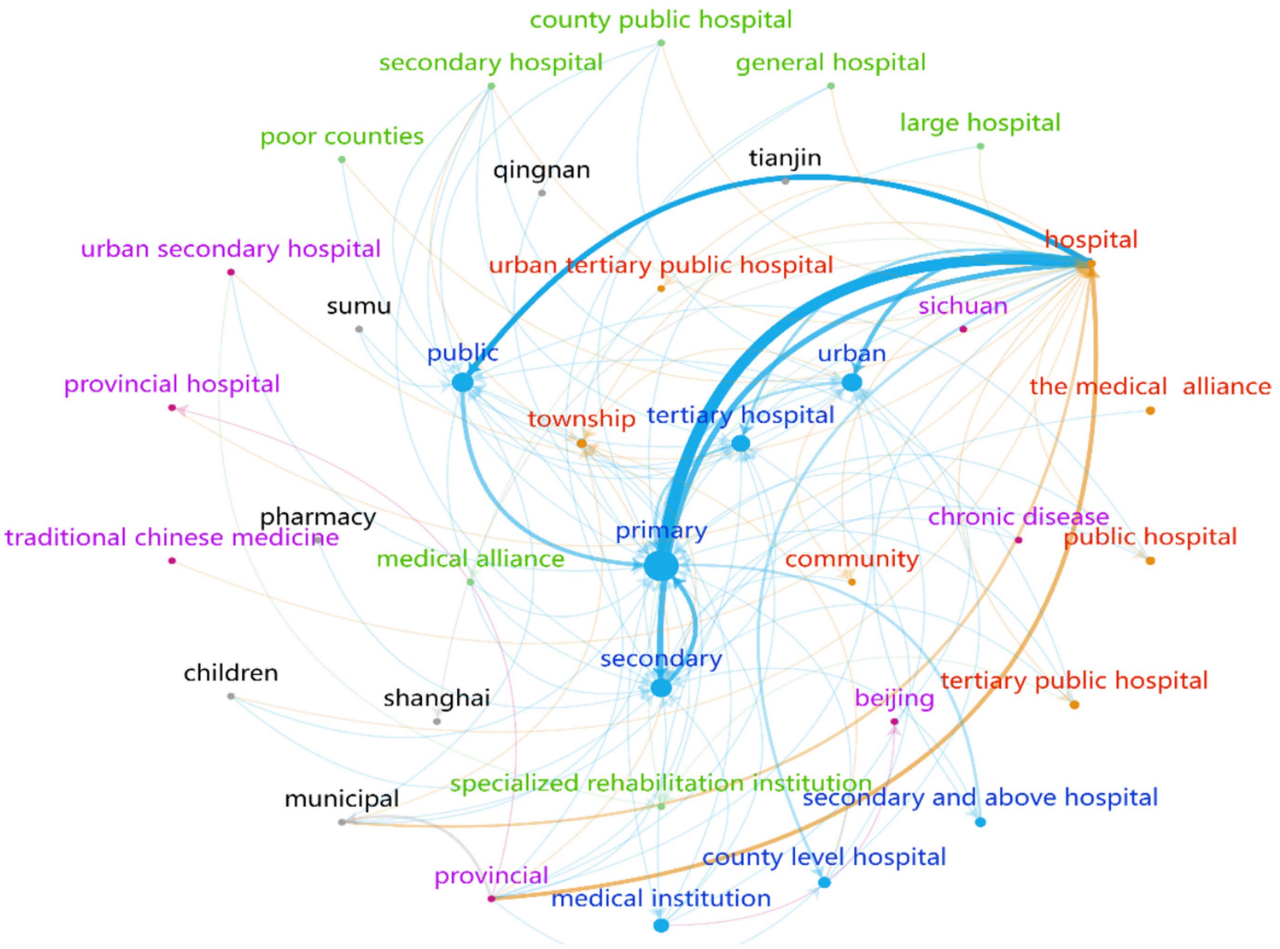
Vertical integration of high-quality medical resources symbiotic unit cooperation network.

To identify the symbiotic units involved in the VI-HQMR, we extracted 482 symbiotic units from 697 policy entries that contained these units, resulting in a symbiotic unit set denoted as U = (U_1_, U_2_, …, U_482_). We counted the word frequency of each symbiotic unit, and multiple symbiotic units appearing within the same policy entry were treated as a single unit to construct the symbiotic unit cooperation network (Figure 4). Each node in the network represents a symbiotic unit, the size of the node corresponds to the word frequency of the unit, indicating how often it appears. The larger the node is, the higher the frequency of occurrence of this symbiotic unit. The lines connecting the nodes represent the correlations between the symbiotic units, with thicker lines indicating stronger correlations. The arrows indicate the direction of the inflow of medical resources.

Among the 482 symbiotic units, the 10 units with the highest word frequency included PHC institutions, hospitals, public hospitals, secondary hospitals, tertiary hospitals, urban hospitals, county hospitals, medical institutions, hospitals at the secondary level and above, and townships. To gain a clearer understanding of the cooperative network among these symbiotic units, this study maintained the distinction between hospital levels and types.

The link between hospitals and PHC institutions is the strongest, with an association frequency of 331 times. This is followed by the connection between public health institutions and hospitals, which has an association frequency of 165 times. The number of associations between primary and secondary health care institutions is 119. These figures indicate a strong cooperative relationship among the three groups of interdependent units.

According to the direction of the arrows in the cooperation network, the majority of arrows point to PHC institutions, secondary and tertiary hospitals, as well as public, urban, and township hospitals. In contrast, fewer arrows are directed towards provincial hospitals, municipal hospitals, and community facilities. Additionally, the VI-HQMR operates in a unidirectional manner, with no closed bidirectional resource flow occurring within the same symbiotic network.

Therefore, the symbiotic unit of VI-HQMR consists of medical institutions at all levels. Public hospitals at the secondary level and above serve as the primary outflow of high-quality medical resources, while primary medical care institutions and rural areas are the main inflow sources of high-quality medical resources. It is important to note that vertical integration is typically a gradual process, transitioning from provincial to municipal to county-level institutions before reaching primary health care (PHC) facilities, rather than a direct, large-scale shift from the highest-level hospitals in the province to PHC institutions.

#### 3.2.2 Symbiotic model

**Figure 5:**
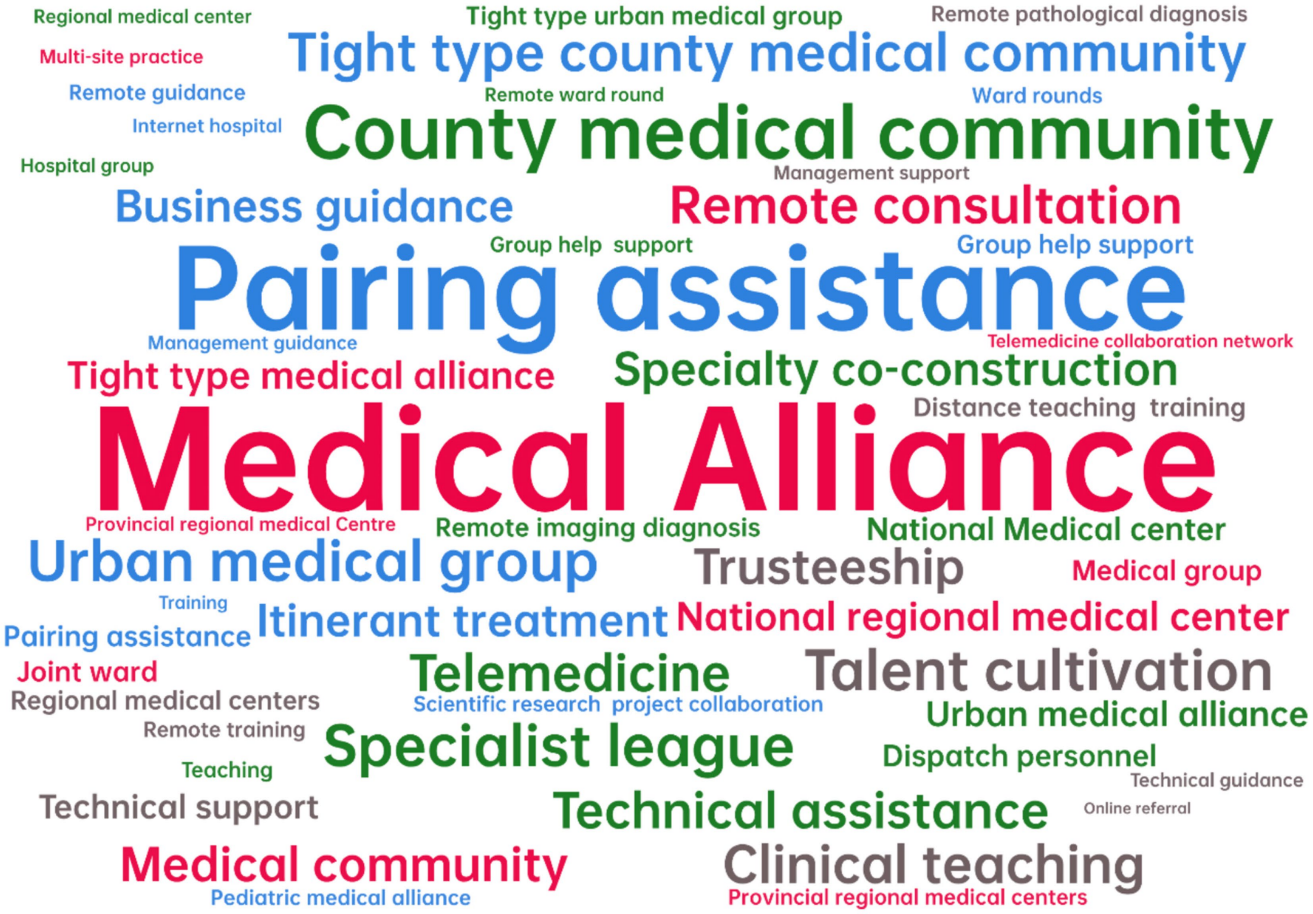
Top 50 symbiotic models of vertical integration of high-quality medical resources.

To explore the connections between the symbiotic units of VI-HQMR, this study first established a symbiotic model based on the connection modes and subsequently refined the final symbiotic model through thematic clustering. The top 50 models with the highest frequency of contact modes were selected to create a word cloud representation of the symbiotic model (Figure 5). In this visualization, larger font sizes indicate higher frequencies of the models. Throughout the VI-HQMR process, the vertical flow of high-quality medical resources has emerged as the most significant method, facilitated by the establishment of medical alliances, pairing assistance, and county medical communities. Specialist league, talent training, urban medical group, clinical teaching, remote consultation, telemedicine, technical cooperation, technical support, trusteeship, specialty co-construction, itinerant treatment, regional medical center, are the means to achieve VI-HQMR. In addition, the ways to establish connections among the symbiotic units also include dispatching personnel, online referral, building joint wards, collaboration on scientific research projects, and management support. Similarly, the symbiotic model reflects the help of high-level medical institutions to poorly resourced primary care sponsor institutions and regions, but does not reflect the benefits of high-level hospitals.

Medical alliance is the main model of VI-HQMR, which refers to the health care organization formed by vertical or horizontal integration of different levels and different categories of medical and health institutions in a certain region. According to the set area, the medical alliance is divided into urban medical group and county medical community. Depending on the degree of close contact, the medical alliance can be classified into tight, semi compact and loose (62) three types. The tight type medical alliance is a kind of operation management mode in which the responsibility and interest community is formed among the health service institutions in the medical alliance, and all personnel and property are unified operation and management. The semi-compact medical alliance refers to an operation management mode formed by the core hospital to sign a contract or agreement with the primary health institution on the basis of the unchanged nature of the assets of the medical service institutions in the medical alliance. Loose medical alliance means that all kinds of health service organizations in the medical alliance only cooperate in medical technology, personnel training, equipment and other aspects. However, various healthcare institutions within the medical alliance are not affiliated with the alliance. The mode of integrating high-quality medical resources at the national level to the provincial level is to establish national or provincial regional medical centers, and the mode of vertically integrating provincial and municipal high-quality medical resources to the city and county level is mainly to establish urban medical groups, while the mode of establishing county medical communities is often adopted in the county level. Symbiosis models such as specialist league, telemedicine, talent training and remote diagnosis and treatment are the embodiment of the above core symbiosis model.

In the symbiosis model of VI-HQMR, the symbiosis organization degree of medical alliance has intermittent symbiosis and continuous symbiosis. Specialist leagues, personnel training, urban medical groups, clinical teaching, remote consultation, telemedicine, technical cooperation, technical support, entrusted management, specialty co-construction, itinerant treatment, and regional medical centers are also dominated by the above two organization degrees.

#### 3.2.3 Symbiotic environment

The symbiotic environment includes four aspects: policy, technology, economy and society. There are 283 policy environments for VI-HQMR, 78 technological environments, 23 economic environments and 0 social environments.

Further summarizing the theme of symbiotic environment, the 283 policy environments contain seven core policies. First, differentiated medical insurance payment policies for medical institutions at all levels. The second is to establish an evaluation index system and take the VI-HQMR as an evaluation index. Third, medical personnel will be allowed to practice in multiple places. Fourth, encourage private hospitals. Fifth, telemedicine and payment policies. Sixth, expand the total number of personnel and smooth channels for promotion. Seventh, promote mutual recognition of inspection results. Seventy-eight technology environments can be summarized as three themes, its telemedicine, informatization and internet hospital. Twenty-three economic environments highlighted financial support from central, provincial and local governments for multiple businesses such as coordinating medical alliances, comprehensive reform of public hospitals, construction of regional medical centers, and upgrading of primary medical service capacity. It is required to set up a special fund for VI-HQMR to support the labor compensation of hospital personnel, training and information construction of supported hospitals. In addition, the economic environment also includes an increase in the total county health insurance budget.

At present, the symbiotic environment supporting the VI-HQMR is dominated by policy environments, and the policy environment is diversified, involving staffing, medical insurance payment, multi-point practice, assessment and evaluation, and performance incentive. The technological environment is more converging, focusing on the application of information means and the establishment of telemedicine collaboration network and Internet hospital. In the economic environment, more emphasis is placed on winning financial support from the central and provincial levels, and the funds for VI-HQMR and business development of the medical community, comprehensive reform of public hospitals, construction of regional medical centers and improvement of PHC capacity are used as a whole. There are few policies mentioning special funds for VI-HQMR.

#### 3.2.4 Symbiotic interface

In the process of VI-HQMR, the symbiotic unit promotes the vertical flow of all elements of medical resources as a whole or a single element, which is called the symbiotic model, and the purpose of adopting the symbiotic model is called the symbiotic interface. In other words, the symbiotic interface is the final result of the interconnection of symbiotic units. There are 102 symbiotic interfaces in this study. The 20 most important symbiotic interfaces and their occurrence times are selected and shown in Table 1. The frequency of the symbiotic interface of VI-HQMR was 656 times, which was the highest among 102 symbiotic interfaces. The others are the vertical integration of personnel, traditional Chinese medicine resources, nursing resources, chronic disease prevention resources, pediatric medical resources, outpatient number sources, patients, technology, inpatient beds, services, pharmaceutical care, management, experts, maternal and child health resources, rehabilitation medical resources, information, critical medical resources, drugs and health management services.

**Table 1:** Top 20 symbiotic interfaces of vertical integration of high-quality medical resources.

Symbiotic interface	Frequency
Medical resource	660
Personnel	49
Traditional Chinese medicine resource	36
Nursing resource	17
Resource for chronic disease prevention and control	16
Pediatric department medical resource	14
Clinic number source	14
Patient	10
Technology	9
Inpatient bed	9
Service	7
Pharmaceutical service	6
Management	6
Expert	6
Maternal and child health service	5
Rehabilitation medical resources	3
Information	3
Critical care medicine resource	3
Drug	2
Health management service	2

**Figure 6:**
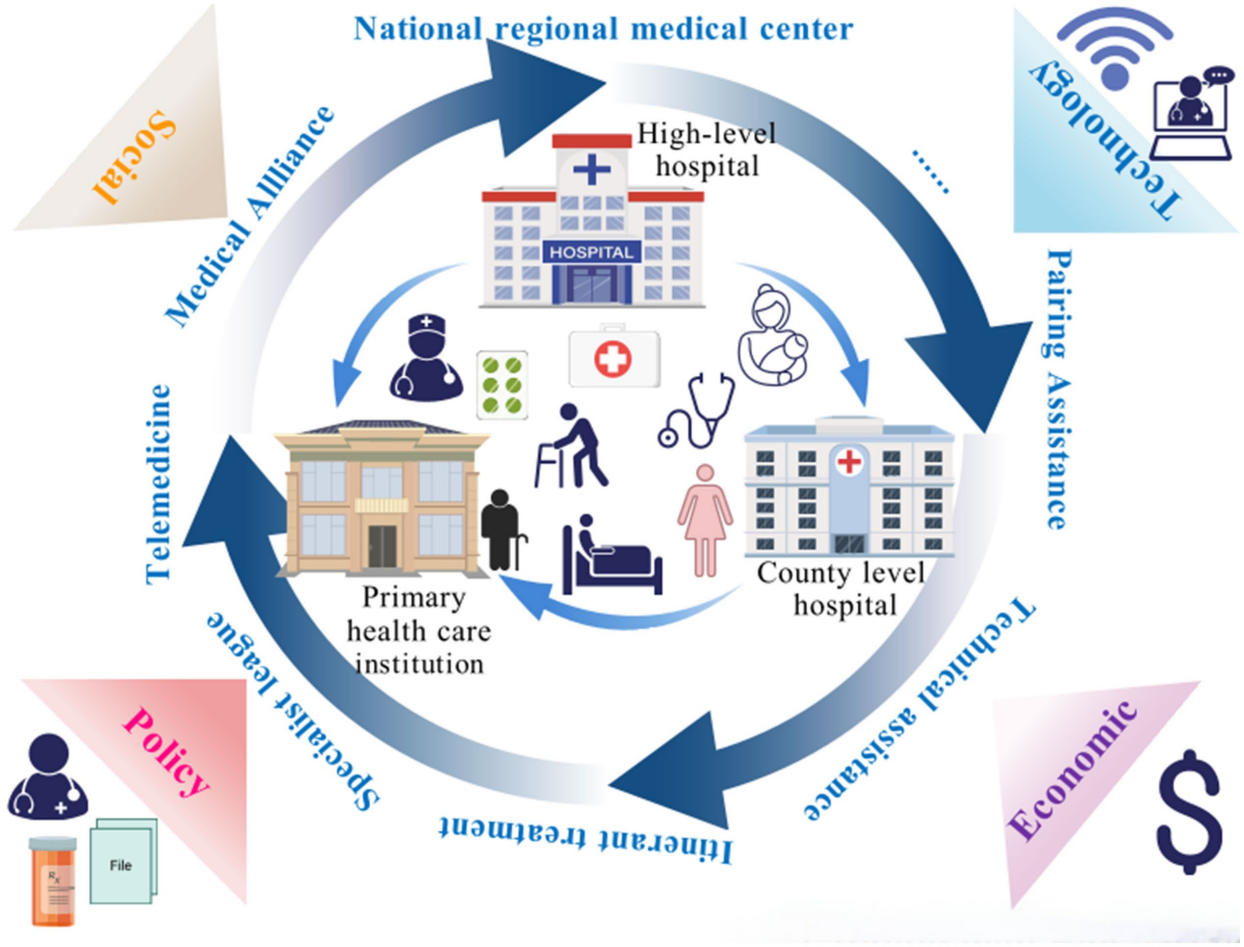
The theoretical model of vertical integration of high-quality medical resource.

This study sorted out the policies on the VI-HQMR, analyzed the internal logic from the perspective of symbiosis theory, and summarized 482 symbiotic units, 549 symbiosis modes, 383 external symbiotic environments and 96 symbiotic interfaces involved in the VI-HQMR, and further summarized them based on the actual work. Based on this, a theoretical model of VI-HQMR was developed (Figure 6). The internal logic of VI-HQMR is to rely on urban higher level hospitals, with the help of policy, technology and economic environments, through the construction of medical alliances, pairing assistance, county medical communities, specialist leagues, personnel training and other ways. Gradually, the elements of high-quality medical resources concentrated at the provincial and municipal levels will be allocated to the municipal and county-level medical institutions and PHC institutions. These factors mainly include personnel, traditional Chinese medicine resources, nursing resources, chronic disease prevention resources, pediatric medical resources and outpatient number sources.

## 4 Discussion

VI-HQMR is an important measure to promote the balanced development of medical and health services, and is of great significance to improving the health of the whole people and promoting the sustainable development of the global health system. China’s medical and health system reform has entered the stage of examining the results, and the VI-HQMR is the only way to improve the primary medical care capacity and further improve the medical and health service system. The main findings of this study are as follows: (i) The VI-HQMR is the result of policy promotion. This work is carried out in the process of medical and health service system planning, medical and health system reform, public traditional Chinese medicine hospital reform, hierarchical diagnosis and treatment, maternal and child health services, medical alliance construction, family doctor contracted services, nursing service resources, and high-quality development of public hospitals. Until the introduction of the first special document in 2024, the VI-HQMR was taken as a separate priority. (ii) The quantity distribution of documents related to VI-HQMR has similar characteristics to the geographical distribution of high-quality medical resources, but it is different in Gansu Province, which is located in the western region. (iii) The symbiotic unit of VI-HQMR is all kinds of medical and health institutions at all levels, secondary and above public hospitals are the main suppliers of high-quality medical resources, and PHC institutions, rural and remote areas are the main importers. (iv) Medical alliance is the main model of VI-HQMR. The symbiotic model is undergoing a change from parasitism to commensalism, and the degree of symbiotic organization is mainly intermittent symbiosis and continuous symbiosis. (v) The main support for VI-HQMR is the policy environment and involves multiple aspects, the technological environment is more integrated, and the economic environment is mainly the financial support of central, provincial and local governments. (vi) In addition to the VI-HQMR, the integration of personnel elements is the main symbiotic interface.

The VI-HQMR has developed from the initial policy point to a special work and introduced implementation rules, which shows that it is crucial in the process of China’s medical and health system reform and the practice of building an integrated delivery system. There are a large number of policy documents introduced in the eastern coastal areas, which reflect the same characteristics as the geographical characteristics of China’s medical resource allocation, which is consistent with the research results of other scholars (63, 64). However, it should be noted that Gansu Province, located in the western region, is relatively short of high-quality medical resources, but the number of policies related to the VI-HQMR introduced by it ranks first in the country. It is speculated that the reason for this phenomenon is the radiation effect of resources and policies. The data of 2020 shows that there are 75 poverty-stricken counties in Gansu Province, 58 of which are the poverty-stricken counties to be supported by the state. The results of this study show that poor counties are also symbiotic units of VI-HQMR, so the promotion of national policies is also an important reason for the introduction of more provincial policies.

As mentioned above, four provinces (municipalities) located in the eastern coastal areas, including Fujian, Zhejiang, Shanghai and Hebei, have made outstanding contributions to the policies related to the VI-HQMR in China, and the relevant policy priorities of the four provinces (municipalities) are different. Fujian focused on the construction of national regional medical centers and the deep integration of county medical communities to promote the flow of technology and management experience to county-level medical institutions. The core measures of Zhejiang are hierarchical diagnosis and treatment policy and technology platform, and guide the VI-HQMR through the implementation of differentiated medical insurance payment government and the creation of “Internet + medical health.” Shanghai promotes VI-HQMR by strengthening community services and building smart communities. The core strategy of Hebei is to promote the VI-HQMR through Beijing-Tianjin-Hebei coordination and Itinerant treatment.

We further discuss the reasons why the policies of VI-HQMR are mainly distributed in the eastern coastal areas, which can be summarized as the pressure of unbalanced resource distribution, the advantages of national pilot policies and the realistic needs of demographic structure changes. First of all, although the eastern coastal areas are economically developed and the total amount of medical resources is rich, there are significant differences within the region. For example, the concentration of tertiary hospitals in Shanghai and Zhejiang is high, but the primary health care capacity is relatively weak. Fujian and Hebei face shortages of mountainous and rural medical resources. This structural contradiction encourages local governments to relieve the pressure of resource allocation through policy innovation. Secondly, the eastern coastal areas have enjoyed more policy dividends of national strategic pilot projects. Fujian is a pilot province for the construction of regional medical centers. Zhejiang is the benchmark of graded diagnosis and treatment reform. There is a national medical center in Shanghai. Hebei benefits from the Beijing-Tianjin-Hebei coordinated development strategy and is supported by Beijing. Finally, China’s rapidly aging population, especially in the eastern provinces, Shanghai has more than 23 percent of the population aged 60 years or older, and the demand for chronic disease management and life-cycle health management has soared, and it is urgent to strengthen primary health care. Therefore, the strong implementation of VI-HQMR in the eastern region is the result of the practical needs to solve the unbalanced distribution of medical resources, the urgent task of coping with the aging population and the national health development strategy.

2017 is an important stage for the intensive introduction of policies related to the VI-HQMR, and it is also a key year for the formation of the framework of China’s basic medical and health care system and the completion of the phased goals of medical reform. Therefore, China has fully launched the pilot work of medical alliance, implemented the hierarchical diagnosis and treatment system, and introduced a number of medical reform policies. As a key task of the reform of the medical and health system, the relevant guidance of the VI-HQMR is integrated into the series of policies of medical reform, and the intensive introduction of relevant documents related to the VI-HQMR in this period is inseparable from the frequent introduction of medical reform policies.

Although the symbiotic units involved in the VI-HQMR include medical institutions at all levels, the outflow of resources is mainly general hospitals and public hospitals. The symbiotic interface summarized in this study shows the vertical integration of various elements of high-quality medical resources, among which personnel, chronic disease prevention resources, traditional Chinese medicine resources, nursing resources, maternal and child health resources are also the key points of VI-HQMR. This requires that high-level specialized medical institutions or superior specialized departments of general hospitals should also participate in it, form a joint force with general hospitals, and promote the effective integration of various elements of high-quality medical resources.

Within a certain geographical scope, the VI-HQMR by establishing medical alliances with hospitals with weak capacity and resources has become an important means to solve the imbalance of medical and health resources allocation and improve the efficiency of PHC services. Under the symbiotic model of medical alliance, the head hospital should provide technical support to the general hospital within its influence, and the general hospital should provide spare medical resources in return (65), the essence of which is mutualism between two or more symbiotic units. However, the symbiotic unit cooperation network constructed in this study shows that the current model among symbiotic units is mostly one-way resource flow of high-quality medical resources and their various elements, and resources often flow from head medical and health institutions to institutions or regions with weak capacity and lack of resources, and a closed loop of two-way resource flow has not been formed within a single symbiotic network, which is still far from the ideal mode of mutualism. In addition, the effect of VI-HQMR in this symbiotic model is also very different because of the closeness and function of the medical alliance. According to the reality of VI-HQMR in various regions, the establishment and application of evaluation index system should be accelerated to evaluate the integration effect, so as to sum up experience and lessons and make timely improvements.

The VI-HQMR is the result of policy promotion. The policy environment occupies a major position in the symbiotic environment, the technological environment is highly integrated as the support of information means and the support of telemedicine service system and Internet hospital, and the economic environment is mainly the financial support of national and provincial levels. The support of social environment to VI-HQMR is not reflected in this study. In China’s decentralized health financing system, the participation of local governments has always been insufficient (66, 67), which is again confirmed in the economic environment of vertical integration of high-quality health care resources. On the one hand, the responsibility of local governments should be compacting, promoting them to establish a compensation mechanism for VI-HQMR, and strengthening their assessment and supervision responsibilities. On the other hand, the construction of telemedicine service system is still in the exploratory stage. In the construction process, we should focus on breaking the dilemma of decentralization and isolation among various systems, and coordinate the deployment of various work in the construction of PHC institutions to establish a multi-functional collaboration system.

In the symbiotic interface, the vertical integration of human resources is an important part of the VI-HQMR. In the policy environment, the establishment, promotion of professional titles, performance incentives are tilted towards the medical staff who assist the PHC institutions, which provides support for the vertical integration of human resources. These benefits for the medical institutions providing assistance can break the pattern of favoritism and symbiosis. Therefore, the key to the transformation of symbiosis model to mutualism is to build a mutually beneficial scene on the symbiotic environment. Both the symbiotic model and the symbiotic interface reflect the vertical integration of maternal and child health resources, traditional Chinese medicine resources, chronic disease prevention resources, rehabilitation medical resources and other clinical specialty resources. It should be noted that although the unbalanced allocation of high-quality medical resources is universal, the allocation of medical resources in different clinical specialties is different from that of medical resources. This requires that when seeking to break through the dilemma of uneven allocation of high-quality medical resources in the region, each region should determine specialties and diseases on the basis of adequate research to ensure that common diseases are given priority to be solved in the region.

Limitations of these findings should be noted. The VI-HQMR is a systematic project, which has many influencing factors. On the one hand, this study aims to explore the internal logic of this work, and this work is a key work led by the national policy, and it is inevitable that the provinces in China will be promoted one after another. Therefore, other factors outside the logical framework of symbiosis theory were not taken into consideration in this study. On the other hand, the results of policy research show that the VI-HQMR is still in the initial stage of exploration, and there is a lack of assessment and evaluation programs for it. Based on this, the study did not evaluate the effectiveness of the work of Chinese provinces (autonomous regions and municipalities directly under the Central Government). Furthermore, this study mainly conducts the analysis based on the explicit content of the policy texts and fails to cover the practical perceptions of various stakeholders during the policy implementation process. However, investigating the viewpoints of stakeholders, establishing an evaluation index system and assessing the effectiveness of this work will be the focus of future research, which will provide more valuable suggestions for improving the VI-HQMR. And in the future, we will consider more larger, multi-center cohorts and longitudinal monitoring to verify the policy implementation effect of the VI-HQMR.

## 5 Conclusion

This study used content analysis to study the policy progress of VI-HQMR in China, and built a theoretical model of VI-HQMR based on symbiosis theory. At this stage, the VI-HQMR is still in the initial stage of exploration. Through the support of symbiotic environment, the symbiotic model is changing from parasitism to commensalism. It is necessary to make efforts in the symbiotic environment to promote the preferential symbiosis model to the optimal mutualism. Health administrative agencies should cooperate with personnel, medical insurance, finance, drug administration, information and other relevant departments to introduce special policies to support the VI-HQMR, and improve the enthusiasm of urban hospitals by means of smooth personnel promotion channels, financial incentives, and information assistance. In addition, to strengthen the supervision of the government at all levels, which directly affect the quality and medical resources longitudinal integration effect.

The medical and health systems of all countries in the world have their own characteristics, but the goal of sustainable development is the common direction of joint efforts. The unbalanced allocation of medical resources hinders health equity, which is also a dilemma faced by both developed and developing countries. Through the policy guidance of VI-HQMR, China will coordinate and integrate it with matching support, medical alliance construction, telemedicine development and hierarchical diagnosis and treatment system, and take multiple measures to solve the problem of unbalanced allocation of high-quality medical resources across and within the region, which can provide some experience for countries in the world to optimize the allocation of high-quality medical resources.

## Data Availability

The original contributions presented in the study are included in the article/supplementary material, further inquiries can be directed to the corresponding author.
